# Epigenome-wide DNA methylation profiling of preeclamptic placenta according to severe features

**DOI:** 10.1186/s13148-020-00918-1

**Published:** 2020-08-24

**Authors:** Ji Hyae Lim, Yu-Jung Kang, Hye Jin Bak, Mi Sun Kim, Hyun Jung Lee, Dong Wook Kwak, You Jung Han, Moon Young Kim, Hyeyeon Boo, Shin Young Kim, Hyun Mee Ryu

**Affiliations:** 1 Center for Prenatal Biomarker Research, CHA Advanced Research Institute, CHA Bundang Medical Center, Seongnam-si, Gyeonggi-do Republic of Korea; 2 Department of Obstetrics & Gynecology, CHA Bundang Medical Center, CHA University, 59, Yatap-ro, Bundang-gu, Seongnam-si, Gyeonggi-do 13496 Republic of Korea; 3 Department of Obstetrics and Gynecology, Ajou University School of Medicine, Suwon, Republic of Korea; 4 Department of Obstetrics & Gynecology, CHA Gangnam Medical Center, CHA University, Seoul, Republic of Korea; 5 Department of Obstetrics & Gynecology, CHA Ilsan Medical Center, CHA University, Pocheon-si, Gyeonggi-do Republic of Korea; 6 Laboratory of Medical Genetics, Medical Research Institute, Cheil General Hospital and Women’s Healthcare Center, Seoul, Republic of Korea

**Keywords:** DNA methylation, Epigenetics, Preeclampsia, Severe features

## Abstract

**Background:**

Preeclampsia (PE) is an obstetric disorder with significant morbidities for both the mother and fetus possibly caused by a failure of the placental trophoblast invasion. However, its pathophysiology largely remains unclear. Here, we performed DNA methylation profiling to determine whether differential patterns of DNA methylation correlate with PE and severe features of PE.

**Materials and methods:**

We extracted DNA from placental tissues of 13 normal, five PE, and eight PE pregnant women with severe features. Genome-wide DNA methylation analysis was performed using the Illumina HumanMethylation 850K BeadChip. New functional annotations of differentially methylated CpGs (DMCs) in PE were predicted using bioinformatics tools.

**Results:**

Significant differences were evident for 398 DMCs, including 243 DMCs in PE and 155 DMCs in PE with severe features, compared with normal placental tissues. Of these, 12 hypermethylated DMCs and three hypomethylated DMCs were observed in both PE groups, thus were independent from severe features. Three hundred seventy-nine DMCs were identified by the presence or absence of severe features. Two hundred genes containing these DMCs were associated with developmental processes and cell morphogenesis. These genes were significantly associated with various PE complications such as disease susceptibility, viral infections, immune system diseases, endocrine disturbance, seizures, hematologic diseases, and thyroid diseases.

**Conclusions:**

This is the first study to investigate the genome-scale DNA methylation profiles of PE placentas according to severe features. The epigenetic variation in the placentas probably resulted in altered developmental processes and immune dysregulation, contributing to PE. This study provides basic information to refine the clinical and pathological mechanisms of the severe features in placenta-mediated PE.

## Background

Preeclampsia (PE) is a hypertensive disorder of pregnancy that affects 2~8% of all pregnancies globally and is one of the leading causes of maternal mortality and morbidity [1]. It is a multisystem disorder characterized by maternal new-onset hypertension and proteinuria after 20 weeks of gestation. In the absence of proteinuria, the finding of new-onset hypertension with maternal organ dysfunction, including thrombocytopenia, renal insufficiency, impaired liver function, and pulmonary edema, is enough to make the diagnosis [1]. Therefore, the clinical phenotype varies, with the signs of the syndrome ranging from increases in blood pressure to more serious complications, including renal and liver dysfunction and seizures. Therefore, recently, it was recommended that the subclassification of PE was according to the presence or absence of severe maternal and fetal features and not according to the severity of symptoms generally known as mild, moderate, or severe [1]. The causes and pathophysiology of PE largely remain a mystery; however, genetic, immunological, endocrine, and environmental factors have all been implicated [2]. Numerous studies have reported alterations of gene expression in various mechanisms related to PE, including trophoblast motility and invasion, angiogenesis, cell adhesion, and immune response [3–7]. Epigenetic events play a major role in these gene expression changes. This would indicate the contribution of epigenetic modifications in the various symptoms and development of PE.

Epigenetic modifications regulate gene expression without changing the DNA sequence. DNA methylation is the most common epigenetic mechanism, which primarily occurs in CpG sites and is critical for optimal placental and fetal development. A few studies investigated the role of global DNA methylation in placentas from pregnancies complicated by PE. These studies have shown that various regions of DNA in the epigenome are hyper- and/or hypo-methylated in PE placentas compared with normal placentas [8–13]. However, there is little research on DNA methylation according to severe features of PE. Therefore, comparative DNA methylation profiling analysis in PE placenta according to severe features may improve our understanding of the pathophysiology of these diseases.

Here, we investigated the epigenome-wide DNA methylation patterns in the placentas of pregnant women with or without severe features of PE and normal pregnant women and identified differentially methylated CpG sites (DMCs). New potential biological functions of genes, including DMCs, were suggested using various bioinformatics tools.

## Results

### Clinical characteristics of the study groups

The clinical characteristics of the study groups are summarized in Table 1. Maternal age, prepregnancy body mass index, gravidity, and blood pressures measured in the first trimester were not different among the three groups. However, the percentage of nulliparous and the highest blood pressures in pregnancy were increased in the two subgroups with PE, compared with the control. Proteinuria was detected in only the PE groups. There were no differences between the two subgroups of PE regarding the highest blood pressures, proteinuria, platelet count, and levels of serum creatinine. Increased levels of liver transaminases were observed in two PE pregnant women with severe features. In pregnancy outcome, the gestational age at delivery was lower in the PE with severe features, compared with the control. Birth weights and fetal growth percentage were also lower in the PE with severe features than in the others. Therefore, the percentage of neonatal intensive care unit admission was higher in the PE group with severe features than in the other groups. The gender ratio of the fetuses was not different among all the study groups.

**Table 1:** Patient demographics and characteristics

Characteristics	Control (*n* = 13)	PE (*n* = 5)	PE with severe feature (*n* = 8)
Maternal age, years	35.7 ± 4.2	37.4 ± 5.0	35.6 ± 2.1
Prepregnancy body mass index, kg/m^2^	24.1 ± 3.5	23.7 ± 4.3	24.8 ± 4.4
Gravidity (*n*)	2.3 ± 0.9	1.8 ± 0.8	1.9 ± 0.6
Nulliparous, %	46.2 (6/13)	60 (3/5)	87.5 (7/8)
Blood pressure and proteinuria			
SBP at booking^a^, mm Hg	111.4 ± 10.9	127.2 ± 10.9	123.9 ± 14.1
DBP at booking^a^, mm Hg	64.9 ± 12.1	78.4 ± 4.2	74.8 ± 8.7
Highest SBP, mm Hg	122.5 ± 8.7	151.2 ± 7.2	155.0 ± 6.7
Highest DBP, mm Hg	72.2 ± 7.4	93.4 ± 6.6	97.3 ± 5.9
Proteinuria (dipstick result)	0	2.4 ± 0.9	1.9 ± 0.4
Pregnancy outcome			
Gestational age at delivery, weeks	38.8 ± 1.2	38.4 ± 1.9	35.8 ± 2.4
Delivery < 37 weeks, %	0 (0/13)	0 (0/5)	62.5 (5/8)
Birthweight, kg	3.3 ± 0.3	3.1 ± 0.5	2.0 ± 0.5
Fetal growth percentage	46.7 ± 6.3	42.4 ± 7.4	5.4 ± 4.1
Neonatal intensive care unit admission, %	0 (0/13)	40 (2/5)	87.5 (7/8)
Male infant, %	46.2 (6/13)	20 (1/5)	62.5 (5/8)

Continuous variables are presented as mean ± standard deviation, and discrete variables are percentages (n/N)

^a^First blood pressure measured in pregnancy, all in the first trimester

### Alteration of DNA methylation in the whole genome of the PE placenta

**Fig. 1:**
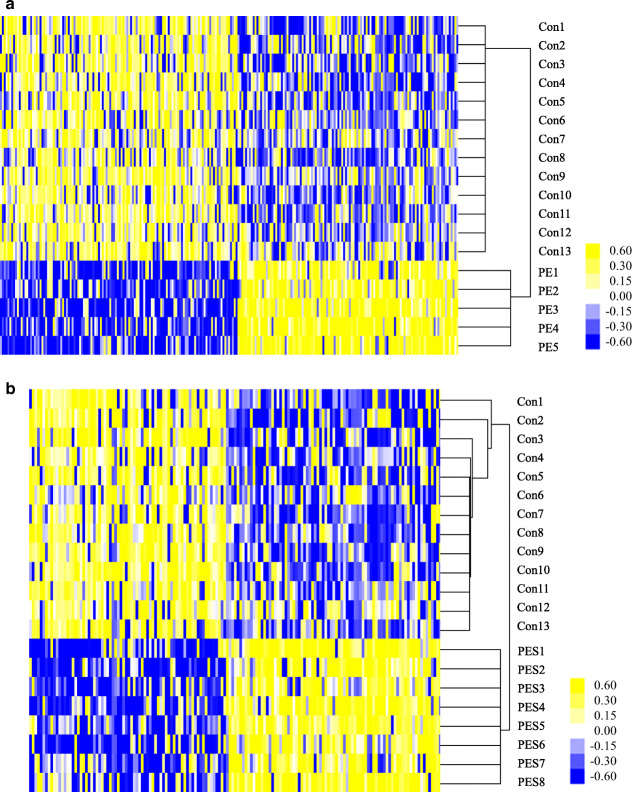
Hierarchical clustering of differentially methylated CpG sites (DMCs) in PE. The methylation degree values from the 850K array were applied to an independent *t* test (*P* < 0.05) and a fold-change criterion (|delta_average of methyl degree| ≥ 0.2). The *P* values were corrected using the Benjamini and Hochberg false discovery rate method to control false positive results from multiple testing. The methylation degree values for these DMCs were subjected to hierarchical clustering. Biological samples are on the x-axis, and DMCs are on the y-axis with strong methylation indicated in the yellow and weak or absent methylation in blue. **a** Control versus PE. **b** Control versus PE with severe features. Con, control. PE, preeclampsia, PES, preeclampsia with severe features

**Fig. 2:**
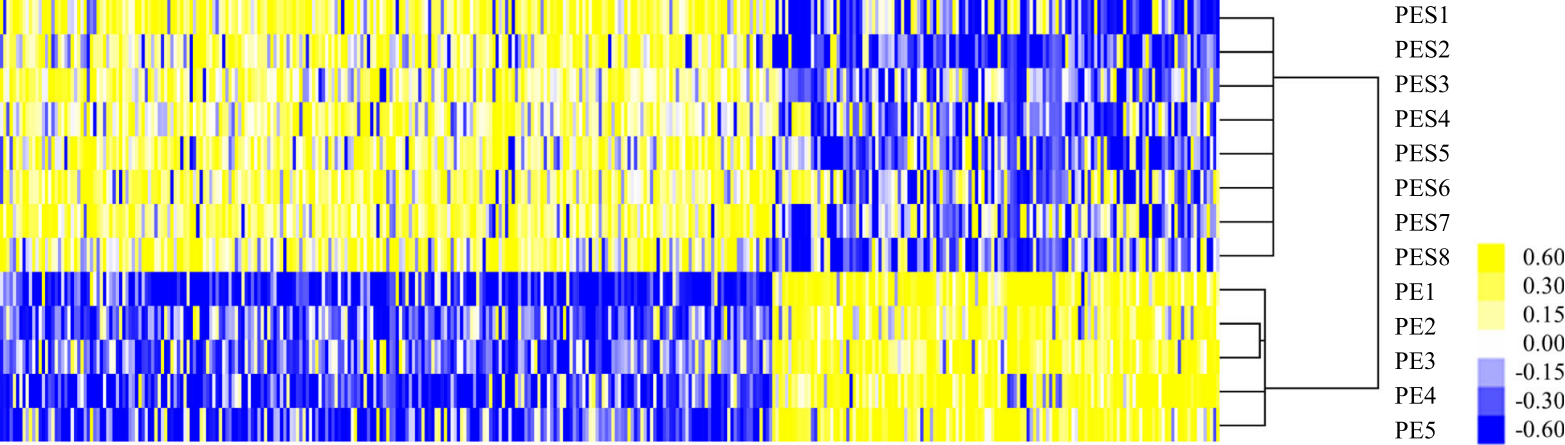
Hierarchical clustering of differentially methylated CpG sites (DMCs) according to severe features of PE. The degree values of methylation from the 850K array were assessed with an independent *t* test (*P* < 0.05) and a fold-change criterion (|delta_average of methyl degree| ≥ 0.2). The *P* values were corrected using the Benjamini and Hochberg false discovery rate method to control false positive results from multiple testing. The degree values of methylation for these DMCs were subjected to hierarchical clustering. Biological samples are on the x-axis and DMCs are on the y-axis with strong methylation indicated in yellow and weak or absent methylation in blue. PE, preeclampsia, PES, preeclampsia with severe features

**Fig. 3:**
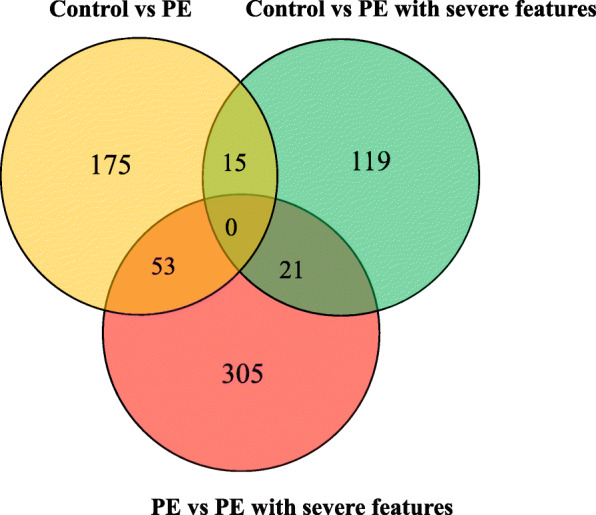
Venn diagram showing the overlap of DMCs with significant changes. PE, preeclampsia

The methylation profiles of placentas were compared separately in three comparison groups: PE versus controls, PE with severe features versus controls, and PE versus PE with severe features. A total of 398 DMCs were identified: 243 in PE (51.9% hypo DMCs, *n* = 126, and 48.1% hyper DMCs, *n* = 117; Additional file 1: Table S1) and 155 in PE with severe features (47.7% hypo DMCs, *n* = 74, and 52.3% hypo DMCs, *n* = 81; Additional file 1: Table S2), as compared with controls (Fig. 1). Among them, 12 hypermethylated DMCs and three hypomethylated DMCs were commonly observed in PE, regardless of the presence or absence of severe features (Additional file 1: Table S1 and Table S2). In the analysis between the two subgroups of PE, significant differences were evident for 379 DMCs, including hypermethylation (*n* = 139, 36.7%) and hypomethylation (*n* = 240, 63.3%) in PE, compared to PE with severe features (Fig. 2). The number of hypomethylated DMCs far exceeded the hypermethylated DMCs (Additional file 1: Table S3). Of these DMCs, most DMCs (80.5%) were specific to the severe features of the disease, and only 74 DMCs (19.5%) were included in 398 DMCs, as compared with controls (Fig. 3). In the distribution of DMCs according to functional genomic regions, there were no differences in the proportion of hypermethylated DMCs and hypomethylated DMCs of most functional genomic regions (Table 2).

**Table 2:** Distribution of DMCs on functional genomic regions

Functional genomic regions	Control versus PE				Control versus PE with severe features				PE versus PE with severe features			
	Hypo DMCs		Hyper DMCs		Hypo DMCs		Hyper DMCs		Hypo DMCs		Hyper DMCs	
	*n*	%	*n*	%	*n*	%	*n*	%	*n*	%	*n*	%
1stExon	3	3.8	0	0.0	1	2.0	1	1.8	5	3.3	8	8.4
3′UTR	2	2.5	3	4.0	0	0.0	3	5.3	8	5.2	0	0.0
5′UTR	10	12.5	7	9.3	9	18.0	9	15.8	27	17.6	13	13.7
Body	51	63.8	43	57.3	28	56.0	30	52.6	96	62.7	47	49.5
TSS1500	11	13.8	16	21.3	11	22.0	13	22.8	13	8.5	15	15.8
TSS200	3	3.8	5	6.7	1	2.0	1	1.8	4	2.6	10	10.5
ExonBnd	0	0.0	1	1.3	0	0.0	0	0.0	0	0.0	2	2.1

*PE* Preeclampsia, *DMCs* Differentially methylated *CpGs UTR* Untranslated regions, *TSS* Transcriptional start site, *TSS200* 0~200 bases upstream of TSS, *TSS1500* 200~1500 bases upstream of the TSS, *Body* between the ATG and stop codon, irrespective of the presence of introns, exons, TSS, or promoters, *ExonBnd* within 20 bases of an exon boundary, i.e., the start or end of an exon

**Fig. 4:**
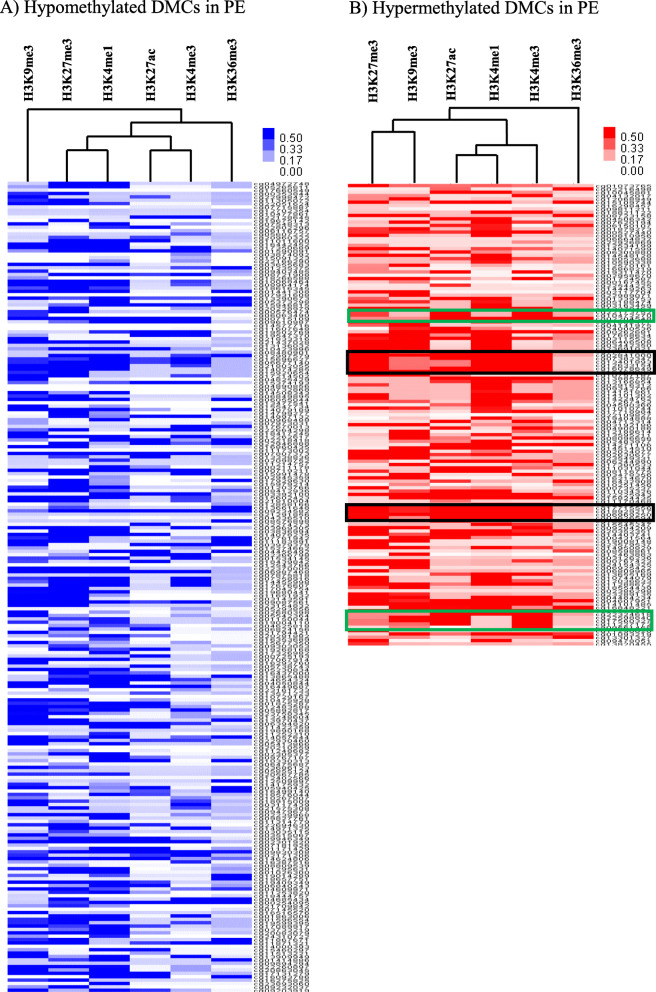
Distribution of differentially methylated CpG sites (DMCs) to PE according to chromatin marks. The scores of chromatin marks for these DMCs were subjected to hierarchical clustering. Chromatin marks are on the x-axis, and DMCs are on the y-axis. **a** Hypomethylated DMCs in PE. **b** Hypermethylated DMCs in PE. Black box: regions of DMCs showing high scores in all marks (H3K27me3, H3K9me3, H3K4me1, H3K4me3, and H3K27ac) except H3K36me3, green box: DMCs of *TICAM2* and *ZNF417*

To further investigate the changes of DNA methylation observed in PE with severe features, we compared the data of the placenta from the Roadmap Epigenomics project [14] with these results. A publicly available dataset of placenta (E091) was reanalyzed to investigate epigenetic regulation between chromatin regions with DMCs (Table 3 and Additional file 1: Table S3). Hypomethylated DMCs in PE had high frequencies in the weak repressed polycomb, repressed polycomb, and heterochromatin regions, whereas hypermethylated DMCs had high frequencies in the regions annotated as weak enhancer, bivalent enhancer, and weak repressed polycomb (Table 3). However, the largest number of DMCs was located in the quiescent/low regions, regardless of the methylation pattern in PE (Table 3). In the distribution of DMCs according to 6 chromatin marks assayed in all epigenome (H3K4me3, H3K4me1, H3K36me3, H3K27me3, H3K9me3, and H3K27ac), hypomethylated DMCs in PE showed enrichment in H3K9me3, H3K27me3, and H3K4me1 throughout the whole genome (Fig. 4). However, hypermethylated DMCs in PE showed enrichment in all marks (H3K27me3, H3K9me3, H3K4me1, H3K4me3, and H3K27ac) except H3K36me3 in the partial regions of chromosome 6 and chromosome 12 (Fig. 4).

**Table 3:** Distribution of DMCs according to chromatin regions of placenta

Chromatin regions	Hypomethylated DMCs in PE, compared with the PE with severe features (*N* = 240)		Hypermethylated DMCs in PE, compared with the PE with severe features (*N* = 139)	
	*n*	%	*n*	%
Active enhancer 1	3	1.3	5	3.6
Active enhancer 2	3	1.3	5	3.6
Active TSS	0	0.0	5	3.6
Bivalent enhancer	8	3.3	10	7.2
Bivalent/poised TSS	6	2.5	1	0.7
Flanking TSS	8	3.3	9	6.5
Flanking TSS downstream	3	1.3	3	2.2
Flanking TSS upstream	3	1.3	4	2.9
Genic enhancer1	0	0.0	1	0.7
Genic enhancer2	2	0.8	1	0.7
Heterochromatin	33	13.8	8	5.8
Quiescent/low	51	21.3	38	27.3
Repressed Polycomb	27	11.3	3	2.2
Strong transcription	8	3.3	2	1.4
Weak enhancer	13	5.4	17	12.2
Weak repressed Polycomb	46	19.2	13	9.4
Weak transcription	14	5.8	10	7.2
ZNF genes and repeats	12	5.0	4	2.9

Additionally, we analyzed associations of PE-related GWAS SNPs with DNA methylation and checked for DMCs around these SNPs [15]. DNA methylation levels of total 51 CpG sites around the SNPs were analyzed (Additional file 1: Table S4). Six CpG sites showed significant differences in DNA methylation levels between three groups (*P* < 0.05 for all, Table 4). However, differences in DNA methylation of the CpG sites did not satisfy the DMC selection criteria (Table 4). Moreover, DNA methylation levels of the CpG sites near PE-related SNPs were not different among all groups (*P* > 0.05 for all, Table 4).

**Table 4:** DNA methylation around PE-related SNPs (GRCh37/hg19 assembly)

Probe ID	MAPINFO	SNP_ID	Control (*n* = 13)	PE (*n* = 5)	PE with severe feature (*n* = 8)	*P*	Bonferroni		
							*P*^a^	*P*^b^	*P*^c^
cg11348599	29,073,223	rs192147271 rs74619608 rs550918608	0.59	0.51	0.53	0.030	0.037	0.358	1.000
cg15188434*	29,105,629	rs192139036	0.69	0.67	0.70	0.794	1.000	1.000	1.000
cg08962087	29,107,120	rs569310504 rs537887549 rs75417109	0.19	0.10	0.15	0.004	0.002	0.362	0.002
cg07027493^#^	29,138,219	rs7320190 rs560703568	0.47	0.48	0.47	0.975	1.000	1.000	1.000
cg10329331^#^	29,139,026	rs545690604 rs18598099 rs113006545	0.97	0.97	0.97	0.831	1.000	1.000	1.000
cg15887927	29,148,952	rs540334005 rs377281528 rs528914368	0.53	0.41	0.50	0.003	0.001	0.001	0.063
cg19371105	29,159,914	rs550630513 rs369261321	0.92	0.84	0.92	0.015	0.002	1.000	0.020
cg18469224	29,160,247	rs537961884	0.95	0.96	0.96	0.044	0.242	0.072	1.000
cg16463452	29,195,249	rs138779088	0.88	0.81	0.89	0.028	0.001	1.000	0.004
cg08931404^†^	29,227,083	rs563719373 rs532541556	0.87	0.88	0.90	0.224	1.000	0.272	0.553

Continuous variables are presented as mean

Kruskal-Wallis test, followed by the Bonferroni correlation for multiple testing

*P*^a^ control versus PE, *P*^b^ control versus PE with severe features, *P*^c^ PE versus PE with severe features

PE-related SNPs: *rs149427560 (MAPINFO: 29,105,870); ^#^rs4769613 (MAPINFO: 29,138,609); ^†^rs12050029 (MAPINFO: 29,227,519)

### Significant influence of DMCs in PE with severe features

In the in silico analysis using 200 genes including the 379 DMCs specific to PE with severe features, system development was the most significant (adj*P =* 0.0016) (Table 5). The largest number of genes (*n* = 70) was involved in the developmental process (adj*P =* 0.0018). In the molecular function category, *CDH13*, *PTPRT*, *CTNNA2*, and *NUMB* were significantly associated with cadherin binding (adj*P =* 0.0114). In the cellular component category, the cell periphery and plasma membrane were significantly associated with the largest number of genes (*n* = 56, both for adj*P <* 0.05), respectively. The disease associations of the genes are shown in Table 6. The most statistically significant associations were for disease susceptibility (adj*P =* 0.00007), and the largest number of genes was involved (*n* = 17). DMC genes were also significantly associated with various PE complications, including viral infections, immune system diseases, endocrine disturbance of nitric oxide synthase, seizures, hematologic diseases, and thyroid diseases (adj*P* < 0.05 for all).

**Table 5:** GO analysis of DMC genes identified according to severe features (GRCh37/hg19 assembly)

	Pathway	Gene symbol	raw*P*	adj*P*
BP	System development	*TRIM10*, *ADAM19*, *ROBO2*, *GAS7*, *HLA-F*, *WNT2*, *RXRA*, *OTX1*, *SLIT3*, *PBX1*, *NRN1*, *CREB5*, *CNTNAP2*, *PPP1R17*, *HLA-DPB1*, *SPTB*, *CTNNA2*, *HLA-DRB1*, *HLA-DPA1*, *FGFR3*, *PLXNA4*, *GPRIN1*, *STK3*, *CBFA2T3*, *ATIC*, *CDH13*, *KRT19*, *ITGA1*, *LSAMP*, *KIRREL3*, *ZFAT*, *MSX1*, *KLK5*, *PRDM16*, *OSTM1*, *CACNA1A*, *ITGA5*, *CD80*, *LOXL2*, *ZIC1*, *BCL11A*, *PCDHB11*, *UBD*, *BAI3*, *PAX8*, *WWOX*, *HLA-DQB2*, *VCAN*, *CPLX2*, *KRT2*, *DAB1*, *NFASC*, *SPP2*, *IKZF1*, *SHOX2*, *HCN1*, *MAGI2*, *IGF2*, *NUMB*, *HOXA13*	2.16e−06	0.0016
	Anatomical structure development	*TRIM10*, *ADAM19*, *SGCZ*, *RXRA*, *OTX1*, *SLIT3*, *PBX1*, *NRN1*, *SPTB*, *CTNNA2*, *HLA-DPA1*, *GPRIN1*, *CDH13*, *KRT19*, *OSTM1*, *LOXL2*, *BCL11A*, *PCDH8*, *UBD*, *HLA-DQB2*, *CPLX2*, *NFASC*, *IKZF1*, *HCN1*, *MAGI2*, *NUMB*, *HOXA13*, *BIN1*, *SPAG16*, *GAS7*, *ROBO2*, *HLA-F*, *WNT2*, *CNTNAP2*, *CREB5*, *PPP1R17*, *HLA-DPB1*, *HLA-DRB1*, *PLXNA4*, *FGFR3*, *STK3*, *CBFA2T3*, *ATIC*, *LSAMP*, *ITGA1*, *KIRREL3*, *ZFAT*, *KLK5*, *MSX1*, *PRDM16*, *CACNA1A*, *ITGA5*, *CD80*, *ZIC1*, *PCDHB11*, *BAI3*, *WWOX*, *PAX8*, *VCAN*, *KRT2*, *DAB1*, *SPP2*, *SHOX2*, *IGF2*	9.46e−06	0.0018
	Developmental process	*TRIM10*, *ADAM19*, *SGCZ*, *RXRA*, *OTX1*, *SLIT3*, *PBX1*, *NRN1*, *SPTB*, *CTNNA2*, *HLA-DPA1*, *GPRIN1*, *CDH13*, *KRT19*, *OSTM1*, *LOXL2*, *EBF3*, *BCL11A*, *PCDH8*, *UBD*, *HLA-DQB2*, *CPLX2*, *NFASC*, *IKZF1*, *HCN1*, *MAGI2*, *NUMB*, *HOXA13*, *BANP*, *BIN1*, *SPAG16*, *GAS7*, *ROBO2*, *JDP2*, *HLA-F*, *GABBR1*, *WNT2*, *CNTNAP2*, *CREB5*, *PPP1R17*, *HLA-DPB1*, *HLA-DRB1*, *PLXNA4*, *FGFR3*, *STK3*, *CBFA2T3*, *ATIC*, *MAPK9*, *LSAMP*, *ITGA1*, *KIRREL3*, *ZFAT*, *KLK5*, *MSX1*, *PRDM16*, *CACNA1A, ITGA5, CD80*, *ZIC1*, *PCDHB11*, *BAI3*, *PAX8*, *WWOX*, *VCAN*, *KRT2*, *DAB1*, *SPP2*, *SPRED3*, *SHOX2*, *IGF2*	9.35e−06	0.0018
	Nervous system development	*ROBO2*, *GAS7*, *WNT2*, *RXRA*, *OTX1*, *SLIT3*, *PBX1*, *NRN1*, *CNTNAP2*, *PPP1R17*, *SPTB*, *CTNNA2*, *FGFR3*, *PLXNA4*, *STK3*, *GPRIN1*, *ITGA1*, *LSAMP*, *KIRREL3*, *MSX1*, *PRDM16*, *CACNA1A*, *ITGA5*, *BCL11A*, *ZIC1*, *PCDHB11*, *PAX8*, *VCAN*, *CPLX2*, *DAB1*, *NFASC*, *IKZF1*, *SHOX2*, *HCN1*, *NUMB*	2.01e−05	0.0018
	Organ development	*TRIM10*, *ADAM19*, *ROBO2*, *HLA-F*, *WNT2*, *RXRA*, *OTX1*, *SLIT3*, *PBX1*, *CREB5, CNTNAP2*, *HLA-DPB1*, *SPTB*, *CTNNA2*, *HLA*, *DRB1*, *HLA-DPA1*, *FGFR3*, *PLXNA4*, *STK3*, *CBFA2T3*, *ATIC*, *KRT19*, *KIRREL3*, *ZFAT*, *MSX1*, *KLK5*, *PRDM16*, *OSTM1*, *CACNA1A*, *CD80*, *LOXL2*, *ZIC1*, *BCL11A*, *UBD*, *PAX8*, *WWOX*, *VCAN*, *HLA-DQB2*, *KRT2*, *DAB1*, *IKZF1*, *SHOX2*, *MAGI2*, *HCN1*, *NUMB*, *HOXA13*	1.53e−05	0.0018
	Single-multicellular organism process	*TRIM10*, *ADAM19*, *OR8G1*, *RXRA*, *OTX1*, *SLIT3*, *PBX1*, *NRN1*, *SPTB*, *CTNNA2*, *HLA-DPA1*, *GPRIN1*, *RIMS1*, *P2RX6*, *CDH13*, *KRT19*, *OSTM1*, *LOXL2*, *TANC1*, *EBF3*, *BCL11A*, *GABRB3*, *PCDH8*, *UBD, HLA-DQB2*, *CPLX2, NFASC, IKZF1*, *HCN1*, *MAGI2*, *NUMB*, *HOXA13*, *BANP*, *BIN1*, *DNAJC5*, *SPAG16*, *GAS7*, *ROBO2*, *HLA-F*, *GABBR1*, *WNT2*, *CNTNAP2*, *CREB5*, *PPP1R17*, *HLA-DPB1*, *HLA-DRB1*, *PLXNA4*, *FGFR3*, *STK3*, *CBFA2T3*, *CHRNB3*, *ATIC*, *LSAMP, ITGA1*, *KIRREL3*, *ZFAT*, *KCNJ16*, *MSX1*, *KCNA6*, *KLK5*, *CACNA1E*, *PRDM16*, *CACNA1A*, *PRKAR1B*, *ITGA5*, *CD80*, *ZIC1*, *DLGAP2*, *PCDHB11*, *BAI3*, *PAX8*, *WWOX*, *VCAN*, *KRT2*, *DAB1*, *SPP2*, *SPRED3*, *SHOX2*, *IGF2*, *CHRNB4*	1.96e−05	0.0018
	Cell morphogenesis involved in differentiation	*MSX1*, *ROBO2*, *CACNA1A*, *WNT2*, *RXRA*, *ITGA5*, *SLIT3*, *LOXL2*, *SPTB*, *CTNNA2*, *BCL11A*, *PLXNA4*, *FGFR3*, *PAX8*, *VCAN*, *DAB1*, *NFASC*, *ITGA1*, *NUMB*, *HOXA13*	1.85e−05	0.0018
	Multicellular organismal development	*TRIM10*, *ADAM19*, *RXRA*, *OTX1*, *SLIT3*, *PBX1*, *NRN1*, *SPTB*, *CTNNA2*, *HLA-DPA1*, *GPRIN1*, *CDH13*, *KRT19*, *OSTM1*, *LOXL2*, *EBF3*, *BCL11A*, *PCDH8*, *UBD*, *HLA-DQB2*, *CPLX2*, *NFASC*, *IKZF1*, *HCN1*, *MAGI2*, *NUMB*, *HOXA13*, *BANP*, *BIN1*, *GAS7*, *ROBO2*, *HLA-F*, *WNT2*, *CNTNAP2*, *CREB5*, *PPP1R17*, *HLA-DPB1*, *HLA-DRB1*, *PLXNA4*, *FGFR3*, *STK3*, *CBFA2T3*, *ATIC*, *LSAMP*, *ITGA1*, *KIRREL3*, *ZFAT*, *KLK5*, *MSX1*, *PRDM16*, *CACNA1A*, *ITGA5*, *CD80*, *ZIC1*, *PCDHB11*, *BAI3*, *WWOX*, *PAX8*, *VCAN*, *KRT2*, *DAB1*, *SPP2*, *SPRED3*, *SHOX2*, *IGF2*	6.54e−06	0.0018
	Cell differentiation	*BIN1*, *TRIM10*, *ROBO2*, *GAS7*, *SGCZ*, *JDP2*, *WNT2*, *GABBR1*, *RXRA*, *SLIT3*, *PBX1*, *CREB5*, *CNTNAP2*, *SPTB*, *CTNNA2*, *FGFR3*, *PLXNA4*, *GPRIN1*, *STK3*, *CBFA2T3*, *MAPK9*, *KRT19*, *ITGA1*, *KIRREL3*, *ZFAT*, *MSX1*, *PRDM16*, *OSTM1*, *CACNA1A*, *ITGA5*, *CD80*, *LOXL2*, *ZIC1*, *BCL11A*, *UBD*, *PAX8*, *WWOX*, *VCAN*, *CPLX2*, *KRT2*, *DAB1*, *NFASC*, *IKZF1*, *MAGI2*, *HCN1*, *HOXA13*, *NUMB*	2.12e−05	0.0018
MF	Cadherin binding	*CDH13*, *PTPRT*, *CTNNA2*, *NUMB*, *HLA-DRB1*, *HLA-DPA1*, *HLA-DQB2*	8.88e−05	0.0114
CC	Synapse part	*BIN1*, *DLGAP2*, *GABRB3*, *DNAJC5*, *PCDH8*, *RIMS1*, *CHRNB3*, *P2RX6*, *GABBR1*, *SYT3*, *PTPRN2*, *TANC1*, *MAGI2*, *CHRNB4*	1.13e−05	0.0004
	Axon	*BIN1*, *DLGAP2*, *ROBO2*, *GABBR1*, *RXRA*, *PTPRN2*, *NFASC*, *CNTNAP2*, *TANC1*, *HCN1*, *KIRREL3*, *CTNNA2*	1.78e−05	0.0004
	MHC class II protein complex	*HLA-DRB1*, *HLA-DPA1*, *HLA-DPB1*, *HLA-DQB2*	1.28e−05	0.0004
	ER to Golgi transport vesicle	*HLA-DRB1*, *HLA-DPA1*, *HLA-DPB1*, *HLA-DQB2*, *HLA-F*	4.01e−05	0.0006
	Clathrin-coated vesicle	*HLA-DRB1*, *BIN1*, *HLA-DPA1*, *DNAJC5*, *HLA-DQB2*, *GABBR1*, *SYT3*, *SH3BP4*, *HLA-DPB1*	0.0002	0.0018
	Cell projection	*BIN1*, *SPAG16*, *ROBO2*, *GAS7*, *CACNA1A*, *GABBR1*, *RXRA*, *ITGA5*, *CNTNAP2*, *TANC1*, *CTNNA2*, *DLGAP2*, *PCDH8*, *GPRIN1*, *WWOX*, *P2RX6*, *CDH13*, *CPLX2*, *PTPRN2*, *NFASC*, *ITGA1*, *HCN1*, *KIRREL3*, *MAGI2*	0.0005	0.0038
	Plasma membrane	*DNAJC5*, *ROBO2*, *HCAR1*, *OR8G1*, *SGCZ*, *OR10R2*, *HLA-F*, *WNT2*, *GABBR1*, *CD1C*, *NRN1*, *CNTNAP2*, *HLA-DPB1*, *SPTB*, *CTNNA2*, *WISP2*, *HLA-DRB1*, *HLA-DPA1*, *FGFR3*, *PLXNA4*, *GPRIN1*, *RIMS1*, *TEX101*, *CHRNB3*, *P2RX6*, *CDH13*, *PTPRT*, *HCAR3*, *KRT19*, *ITGA1*, *LSAMP*, *KIRREL3*, *KCNJ16*, *KCNA6*, *CACNA1E*, *TICAM2*, *TNFRSF10B*, *CACNA1A*, *ITGA5*, *CD80*, *TANC1*, *OR10J5*, *DLGAP2*, *RIMBP2*, *PCDHB11*, *GABRB3*, *PCDH8*, *BAI3*, *WWOX*, *HLA-DQB2*, *PTPRN2*, *NFASC*, *HCN1*, *MAGI2*, *NUMB*, *CHRNB4*	0.0039	0.0170
	Cell periphery	*DNAJC5*, *ROBO2*, *HCAR1*, *OR8G1*, *SGCZ*, *OR10R2*, *HLA-F*, *WNT2*, *GABBR1*, *CD1C*, *NRN1*, *CNTNAP2*, *HLA-DPB1*, *SPTB*, *CTNNA2*, *WISP2*, *HLA-DRB1*, *HLA-DPA1*, *FGFR3*, *PLXNA4*, *GPRIN1*, *RIMS1*, *TEX101*, *CHRNB3*, *P2RX6*, *CDH13*, *PTPRT*, *HCAR3*, *KRT19*, *ITGA1*, *LSAMP*, *KIRREL3*, *KCNJ16*, *KCNA6*, *CACNA1E*, *TICAM2*, *TNFRSF10B*, *CACNA1A*, *ITGA5*, *CD80*, *TANC1*, *OR10J5*, *DLGAP2*, *RIMBP2*, *PCDHB11*, *GABRB3*, *PCDH8*, *BAI3*, *WWOX*, *HLA-DQB2*, *PTPRN2*, *NFASC*, *HCN1*, *MAGI2*, *NUMB*, *CHRNB4*	0.0062	0.0235

*BP* Biological process, *MF* Molecular function, *CC* Cellular component, *rawP P* value from hypergeometric test, *adjP P* value adjusted by the multiple test adjustment

**Table 6:** Disease association of DMC genes identified according to severe features (GRCh37/hg19 assembly)

Disease	Gene symbol	raw*P*	adj*P*
Disease susceptibility	*MSX1*, *RBFOX1*, *GABBR1*, *CNTNAP2*, *TCERG1L*, *HLA-DPB1*, *HLA-DRB1*, *SORCS1*, *GABRB3*, *ADGRB3*, *CHRNB3*, *CSMD1*, *MAGI2*, *ZFAT*, *FAM69A*, *CHRNB4*, *PSORS1C1*	3.69e−07	7.63e−05
Viral infections	*KLK5*, *WNT2*, *TNFRSF10B*, *ITGA5*, *CD80*, *LOXL2*, *WISP2*, *FGFR3*, *CBFA2T3*, *WWOX*, *PAX8*, *KRT19*, *IKZF1*, *IGF2*	0.0002	0.0016
Immune system diseases	*HLA-F*, *CD80*, *HLA-DPB1*, *HLA-DPA1*, *HLA-DRB1*, *ATIC*, *HLA-DQB2*, *PTPRN2*, *RNF39*, *IKZF1*, *INS-IGF2*	0.0004	0.0023
Endocrine disturbance NOS	*KLK5*, *BCL11A*, *HLA-DRB1*, *WWOX*, *PAX8*, *PTPRN2*, *KRT19*, *INS-IGF2*, *IGF2*	0.0002	0.0016
Seizures	*DNAJC5*, *GABBR1*, *CACNA1A*, *CNTNAP2*, *GABRB3*, *HCN1*	0.0006	0.0031
Hematologic diseases	*PRDM16*, *SPTB*, *BCL11A*, *HLA-DRB1*, *FGFR3*, *IKZF1*	0.0037	0.0086
Thyroid diseases	*HLA-DRB1*, *PAX8*, *KRT19*, *INS-IGF2*, *ZFAT*	0.0003	0.0020

*rawP P* value from hypergeometric test, *adjP P* value adjusted by the multiple test adjustment

**Fig. 5:**
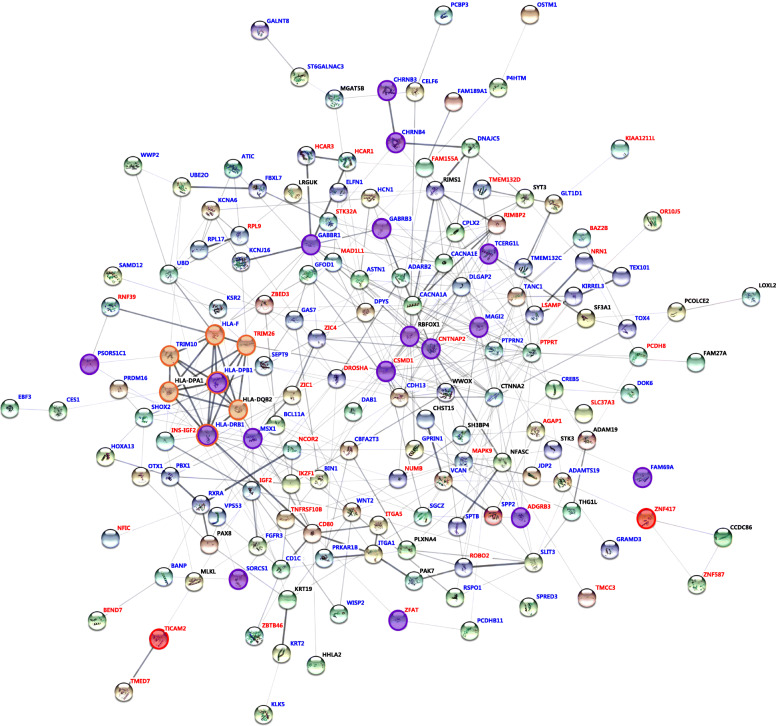
Interaction networks of DMCs according to severe features of PE. The list of the genes identified was subjected to STRING (v. 11.0) analysis to reveal functional interactions. Each node represents a protein, and each edge represents an interaction. Thicker lines represent stronger associations. Red letters and blue letters present hypermethylated and hypomethylated genes, respectively, in PE, compared to PE with severe features. Purple and orange circles represent genes that are related to disease susceptibility and IFNγ-mediated signaling pathway, respectively. Red circles represent *TICAM2* and *ZNF417* containing DMCs specific to severe features of PE

The dynamic complex of signaling was made up of 159 of 200 genes and showed 424 interactions under a confidence score of 0.25 (Fig. 5). Protein-protein interaction enrichment was statistically significant (*P* < 1.0e−16). The interacting genes consisted of 45 hypermethylated (red), 88 hypomethylated (blue), and 26 included both hypermethylated and hypomethylated regions (black) in PE, compared to PE with severe features. Various hypermethylated genes act as connecting nodes in the dynamic complex of hypomethylated genes. All 17 genes that are related to disease susceptibility were included in the complex (purple circles). Among them, *HLA-DPB1* and *HLA-DRB1* were cluster members of interferon-gamma (IFNγ)-mediated signaling pathway related to PE (FDR = 0.0222, orange circles).

### Validation of microarray analysis by methylation-specific quantitative real-time PCR

To verify the microarray results of DMCs, we selected DNA regions, including two or more consecutive DMCs and MSRE recognition site. Of these, the hypermethylated DMC regions of *HIST1H3E* in PE, regardless of the disease’s severe features and the regions of *TICAM2* and *ZNF417*, which constitute an interaction network based on DMCs specific to the severe features of PE, were finally selected and then confirmed their DNA methylation patterns using methylation-specific quantitative real-time PCR.

The ΔCt value of *HIST1H3E* was significantly lower in both PE subgroups than controls. This indicates that the gene was significantly hypermethylated in both PE and PE with severe features, compared with those in controls. Of the DMCs specific to severe features of PE, the ΔCt values of *TICAM2* and *ZNF417* were significantly lower in the PE than the PE with severe features (*P* < 0.05 for both). The methylation patterns of the DNA regions were consistent with that of the array (Table 7).

**Table 7:** Ct values of genes using methylation-specific quantitative real-time PCR

Characteristics of DMCs	Target gene	Method	Control	PE	PE with severe feature	P*	Bonferroni		
							*P*^a^	*P*^b^	*P*^c^
DMCs commonly changed in PE, regardless of severe features	*HIST1H3E*	Methyl degree of array	0.201	0.418	0.481	0.001	0.002	0.007	0.590
		ΔCt of MSRE-qPCR	4.895	3.638	2.485	< 0.001	0.025	< 0.001	0.056
DMCs specific to severe features of PE	*TICAM2*	Methyl degree of array	0.336	0.511	0.286	< 0.001	< 0.001	0.02	< 0.001
		ΔCt of MSRE-qPCR	3.112	2.308	3.231	0.045	0.026	0.736	0.038
	*ZNF417*	Methyl degree of array	0.298	0.452	0.199	< 0.001	< 0.001	0.006	< 0.001
		ΔCt of MSRE-qPCR	4.886	3.193	5.811	0.038	0.082	0.312	< 0.001

Data are expressed as means

*Kruskal-Wallis test, followed by the Bonferroni correlation for multiple testing

*P*^a^ control versus PE, *P*^b^ control versus PE with severe features, *P*^c^ PE versus PE with severe features, *PE* preeclampsia, *Ct* cycle threshold, *MSRE-qPCR* real-time quantitative PCR using methylation-specific restriction enzyme

## Discussion

The mechanisms by which pregnancy triggers or aggravates hypertension remain unsolved. Indeed, hypertensive disorders remain among the most significant and intriguing unsolved problems in obstetrics. Of hypertensive disorders, PE is a syndrome in which hypertension is only one important aspect. Maternal and fetal genes may have independent or interactive effects on the risk of PE. The heterogeneous nature of the disorder, with a sliding scale of severe features, has resulted in differences in the definition of PE. Accumulating evidence suggests that PE, including various pathological changes, occur by alterations of the complex regulation of many genes [8–13]. The alterations could affect the roles of the upstream effector genes and the downstream target genes that are affected by DNA methylation, which are distributed throughout the genome of PE individuals. Therefore, investigating the epigenomic changes that contribute to the various complications of PE may improve understanding of its pathophysiology.

In the current study, we performed DNA methylation profiling in the placentas of normal, PE, and PE pregnant women with severe features. We found that DNA hypomethylation across the genome was evident in PE, compared to PE with severe features. In other words, the dominance of hypermethylated DMC was presented in the PE with severe features. Moreover, our result showed that the patterns of hypomethylated DMCs or hypermethylated DMCs in PE were different according to the regions and marks of chromatin. In a functional annotation analysis of these DMC genes, they were significantly related to the various clinical complications and pathophysiology that were associated with severe features of PE. This finding suggests the possibility of epigenetic therapy by inhibiting global DNA hypermethylation in PE with severe features. Furthermore, this could lead to the development of potential applications for a clinical detection test of PE with severe features.

Eukaryotic organisms package their genetic material into chromatin, generating a physical barrier for transcription factors (TFs) to interact with their cognate sequences. The ability of TFs to bind DNA regulatory elements is modulated by changes in the chromatin structure, including histone modifications, histone variants, ATP-dependent chromatin remodeling, and the methylation status of DNA [16, 17]. Polycomb group (PcG) is one among groups of chromatin regulatory genes and generally contribute to the maintenance of the repressed state through the concerted action of polycomb repressive complexes [16, 18, 19]. In the early embryo, complexes composed of specific sets of PcG proteins are known to assist in structuring chromatin by interpreting the silent or active chromatin state [20–24]. Enhancers are DNA-binding elements characterized by highly sophisticated and various mechanisms of action allowing for the specific interaction of general and tissue-specific TFs [17, 25–27]. In this study, we found that hypomethylated DMCs in PE were mainly distributed in the repressed polycomb regions, whereas hypermethylated DMCs in PE were mainly distributed in the enhancer regions. Moreover, the distribution of DMCs according chromatin marks showed a considerable discrepancy, according to their methylation patterns. These findings suggest the possibility of a comprehensive epigenetic control between chromatin regions and DMCs in the pathophysiology of PE.

In this study, the methylation degree of *HIST1H3E*, *TICAM2*, and *ZNF417* genes were confirmed by methylation-specific real-time PCR. The results were consistent with those of the microarray analysis. Among them, *HIST1H3E* appeared to have an important potential in PE. It encodes a replication-dependent histone that is a member of the histone H3 family. Transcripts contain a palindromic termination element with methylation sites. Among its related pathways, activated PKN1 stimulates the transcription of androgen receptor-regulated genes *KLK2* and *KLK3* and cytokine signaling in the immune system. It was recently reported that PE with severe features was associated with alterations in cytotrophoblasts of the smooth chorion and gene expression of *HIST1H3E* was significantly decreased in PE with severe features than in control [28]. However, the precise mechanism by which *HIST1H3E* is regulated by its interaction with PE has yet to be determined. In this study, we found that the TSS1500 region of *HIST1H3E* is hypermethylated in PE regardless of the presence or absence of severe features. This epigenetic change of *HIST1H3E* in PE may provide additional insight into the pathophysiology of PE generated by its misregulation. Additionally, *TICAM2* on chromosome 5 is the protein-coding gene of the TIR domain-containing adapter molecule 2. Among the pathways related to *TICAM2* are TLR4 signaling and RIG-I/MDA5-mediated induction of IFN-alpha/beta pathways. TLR4 signaling is activated in the placenta of women with PE. The TLR4-mediated immune response at the maternal-fetal interface contributes to poor early placentation and may culminate into a PE-like syndrome. In a previous study investigating ATP-binding cassette transporter expression in the human placenta as a function of pregnancy, gene expression of *TICAM2* was upregulated in the placentas of women who delivered preterm compared with those women who delivered at term [29]. In this study, PE with severe feature was accompanied with preterm delivery. We found hypomethylation of the *TICAM2* in PE with severe feature of preterm delivery but not in PE with delivery at term. This indicates the possibility of an epigenetic effect in the activation of the gene expression in PE with preterm delivery. In this study, there were more incidents of *ZNF417* hypomethylation in PE with severe features than in PE. In a previous study of DNA methylation profiles in PE and healthy control placentas, *ZNF417* was included in differentially methylated genes between PE cases and controls and associated with DNA binding and transcription regulation [30]. In the study, a total of eight PE cases included six with preterm delivery and two with term delivery [30]. Due to the limited number of cases, it would have been difficult to subclassify the severe features of the disease. Their results probably reflected the data of PE with preterm delivery. We found that *ZNF417* was hypomethylated in PE with severe features than in PE. Therefore, our result suggests that the difference in the degree of DNA methylation in *ZNF417* may be widened according to the severe features of the disease.

Additionally, we predicted the dynamic signaling complex of genes, including the DMCs specific to PE with severe features and found that all genes that are related to disease susceptibility were included in the complex. Among them, *HLA-DPB1* and *HLA-DRB1* were confirmed as members in a cluster of IFNγ-mediated signaling pathway related to PE. Human leukocyte antigens (HLA) are necessary for immune recognition, acceptance, and rejection of transplanted organs and tissue grafts, as well as maternal-fetal immune tolerance [31, 32]. These features are known to protect the fetal allograft from maternal immune rejection. HLA-DR and HLA-DP are major histocompatibility complex (MHC) class II molecules, and their constitutive expression is restricted to antigen-presenting cells, such as dendritic cells, B lymphocytes, macrophages, and thymic epithelial cells. MHC class II expression can be induced in most cell types by IFNγ [33–35]. In prior studies of *HLA-DPB1* genotype in severe PE, the association between homozygosity of the *HLA-DPB1* and severe PE is still controversy [36, 37]. In this study, we firstly found that *HLA-DPB1* and *HLA-DRB1* were hypermethylated in PE with severe features compared with PE. These epigenetic changes may suggest a potential association between disease susceptibility and IFNγ-mediated signaling in PE with severe features. Therefore, these in silico results could provide candidate genes based on DMCs for further experimental validation perhaps integrating more functional annotations.

Several studies have compared the DNA methylation profiles between normal and PE patients and have demonstrated common epigenetic characteristics [8–13]. This DNA methylation pattern may be tissue- and developmental stage-specific in PE. Moreover, PE as a heterogeneous syndrome may involve continuous epigenetic changes in the whole genome. These epigenetic changes may be the basis of severe features in PE. Therefore, it is important to investigate epigenetic changes by the severe features of PE to clarify the pathogenesis of PE. They were significantly associated with disease susceptibility as well as various PE complications, viral infections, immune system diseases, endocrine disturbance of NOS, seizures, hematologic diseases, and thyroid diseases. Our results suggest an epigenetic contribution to the various pathophysiology of PE. However, many of our results were based on results using the bioinformatics tools of databases. The in silico results are not strong enough to justify the functional significance of genes. These potential pathways with epigenetic changes in PE remain to be further elucidated. Furthermore, this study was limited by its small sample size and no adjustment for confounders. Therefore, future, larger studies in new cohorts are needed to substantiate our findings. Despite the limitations of this study, this is the first study to analyze epigenetic changes according to the disease’s severe features. We found various DMCs of whole genome in placenta according to the severe features of PE. Our findings warrant further studies addressing the DNA methylation of the whole genome associated with the pathogenesis of severe features in PE.

## Conclusions

In conclusion, based on our data, DNA methylation changes across the genome in PE with severe features may play a key role in the pathophysiology of disease’s various forms and could seem to detect the progression of severe features. Further studies may support the functional significance of our epigenetic insight into the pathophysiology of PE.

## Material and methods

### Study subjects

This study was approved by the Institutional Review Board (IRB) and the Ethics Committee of Cheil General Hospital (#CGH-IRB-2017-22). Singleton pregnant women who attended antenatal care at the hospital’s Department of Obstetrics and Gynecology between August 2010 and August 2017 were enrolled in this study. Written informed consent was obtained from all participants before the collection of samples and subsequent analysis.

Genome-scale DNA methylation in the placenta was compared in three groups of pregnancies: PE (*n* = 5), PE with severe features (*n* = 8), and controls (*n* = 13). PE was defined as hypertension (systolic blood pressure, SBP ≥ 140 mmHg and/or diastolic blood pressure, DBP ≥ 90 mmHg on at least two occasions 4 h apart) and proteinuria (≥ 300 mg in a 24 h urine collection specimen and/or ≥ 1+ on dipstick testing) after 20 weeks of gestation. PE was subcategorized as “PE” and “PE with severe features” according to the criteria [38]. The severe features of PE were defined as DBP ≥ 110 mmHg, SBP ≥ 160 mmHg, onset before 34 weeks of gestation, or a birth weight below the 10th percentile based on gender and gestational age at birth. Control cases were defined as women without medical and obstetric complications that presented for delivery at term (≥ 37 weeks of gestation). Immediately after delivery (≤ 30 min), placental biopsies were collected from the fetal side of the placenta. These samples (1 g) were rinsed in phosphate-buffered saline to remove any contamination with maternal blood and amniotic fluid, snap-frozen in liquid nitrogen, and stored at − 80 °C until required.

### Genome-scale DNA methylation microarray

Genomic DNA was extracted from placental tissue with a QIAamp Tissue Kit (Qiagen, Hilden, Germany) according to the manufacturer’s protocol. Placental DNA underwent sodium bisulfite conversion using an EZ DNA Methylation Kit (Zymo Research, Irvine, CA, USA). The bisulfite-converted DNA (200 ng) was hybridized to an Illumina HumanMethylationEPIC BeadChip (Illumina, Inc., San Diego, CA, USA) (850K array), which provides genome-wide coverage containing > 850,000 CpG methylation sites per sample. Amplification, hybridization, washing, labeling, and scanning of the 850K array were performed by Macrogen (Seoul, Korea).

The raw data were extracted as *β* values for each CpG for each sample with the R watermelon package. The *β* values were calculated by subtracting background using negative controls on the array and taking the ratio of the methylated signal intensity to the sum of both methylated and unmethylated signals. The *β* values of 0 to 1 were reported for each CpG site, which is related to the percentage of methylation, from 0 to 100%. As a quality control step for Illumina array data analysis, we eliminated probes with a detection *P* value > 0.05 in Student’s *t* test in any sample. Probes that mapped to the sex chromosomes and/or known single nucleotide polymorphisms were removed from the analysis.

The DMCs between groups were identified based on the average DNA methylation level difference (delta beta, Δβ) comparison and significance analysis [39]. The final set of candidate genes was constituted by false discovery rates (FDR) ≤ 0.05, Δβ > 0.2 (indicating > 20% difference in DNA methylation), and a *P* value < 0.05 by Student’s *t* test.

### Functional annotation analysis

To investigate the chromatin regions of the DMCs, the dataset of fetal placenta (E091) from the Roadmap Epigenomics project (https://egg2.wustl.edu/roadmap/web_portal/) was used. A publicly available dataset of fetal placenta was reanalyzed to investigate a more comprehensive panoramic of epigenetic regulation between chromatin regions with DMCs. In addition, we analyzed association between PE-related GWAS SNPs [14] with DNA methylation. The windows around each SNP were created based on their coordinates. DNA methylation degrees of CpG sites in the windows were analyzed.

The lists of genes with DMCs were submitted to a functional annotation tool provided by WebGestalt (http://www.webgestalt.org/webgestalt_2013/). Gene ontology (GO) analysis and disease-associated gene analysis were performed according to the criteria of a statistic hypergeometric test as prior study [40]. The Search Tool for the Retrieval of Interacting Genes (STRING v. 11.0) database was used to predict an interactive network of candidate genes. We constructed an interactive network of candidate genes with a confidence score of 0.25.

### Methylation-specific quantitative real-time PCR of DMCs

We confirmed the methylation level of the 850K array by real-time PCR using methylation specific restriction enzyme (MSRE) [40]. Samples that had been used for the 850K array were used for methylation-specific quantitative real-time PCR. The sequences of PCR primers used and PCR conditions are presented in Additional file 1: Table S5. For the analysis of the DMC methylation levels, the delta (Δ) threshold cycle (Ct) value was calculated as ΔCt = Ct_MSRE_ − Ct_input_. The smaller the ΔCt value, the higher the methylation level of a target gene.

### Statistical analysis

Descriptive data are presented as means with standard deviation and categorical variables as proportions and counts. The methylation levels in the study groups were compared using Kruskal-Wallis tests, followed by the post hoc Bonferroni correction test for multiple comparisons, and the Mann-Whitney *U* test for comparisons between the two groups [41]. Values of *P* < 0.05 were considered statistically significant. Statistical analyses were performed with the Statistical Package for the Social Sciences version 25.0 (SPSS Inc. Chicago, IL, USA). The statistical power of this study was calculated using post hoc analysis of the G*Power program 3.1.9.2 (Heinrich-Heine-Universität, Dusseldorf, Germany) as our previous studies [42, 43]. Based on an effective size of 0.8, the sample size used in our study had > 90% power at an α error of 0.05 with two-tails.

## Supplementary information

**Additional file 1: Table S1**. Identified DMCs in PE, as compared with the controls. **Table S2**. Identified DMCs in PE with severe features, as compared with the controls. **Table S3**. Identified DMCs in PE, as compared with the PE with severe features. **Table S4**. DNA methylation of CpG sites around PE-related GWAS SNPs. **Table S5**. Primer sequences and PCR condition for methylation specific quantitative real-time PCR.

## Data Availability

Not applicable.

## Abbreviations

Ct: Threshold cycle
DMCs: Differentially methylated CpG sites
GO: Gene ontology
HLA: Human leukocyte antigens
IFNγ: Interferon-gamma
MHC: Major histocompatibility complex
MSRE: Methylation-specific restriction enzyme
PcG: Polycomb group
PE: Preeclampsia
TFs: Transcription factors
